# Numerical Investigations on Electric Field Characteristics with Respect to Capacitive Detection of Free-Flying Droplets

**DOI:** 10.3390/s120810550

**Published:** 2012-08-03

**Authors:** Andreas Ernst, Klaus Mutschler, Laurent Tanguy, Nils Paust, Roland Zengerle, Peter Koltay

**Affiliations:** 1 Laboratory for MEMS Applications, Department of Microsystems Engineering (IMTEK), University of Freiburg, 79110 Freiburg, Germany; E-Mails: klaus.mutschler@imtek.de (K.M.); laurent.tanguy@imtek.de (L.T.); nils.paust@hsg-imit.de (N.P.); roland.zengerle@imtek.de (R.Z.); peter.koltay@imtek.de (P.K.); 2 Biofluidix GmbH, Georges-Koehler-Allee 103, 79110 Freiburg, Germany

**Keywords:** droplet sensor, CFD simulation, capacitive measurement, multi physics simulation, non-contact sensor, online process control

## Abstract

In this paper a multi-disciplinary simulation of a capacitive droplet sensor based on an open plate capacitor as transducing element is presented. The numerical simulations are based on the finite volume method (FVM), including calculations of an electric field which changes according to the presence of a liquid droplet. The volume of fluid (VOF) method is applied for the simulation of the ejection process of a liquid droplet out of a dispenser nozzle. The simulations were realised using the computational fluid dynamic (CFD) software CFD ACE+. The investigated capacitive sensing principle enables to determine the volume of a micro droplet passing the sensor capacitor due to the induced change in capacity. It could be found that single droplets in the considered volume range of 5 nL < V_drop_ < 100 nL lead to a linear change of the capacity up to ΔQ < 30 fC. The sensitivity of the focused capacitor geometry was evaluated to be S_i_ = 0.3 fC/nL. The simulation results are validated by experiments which exhibit good agreement.

## 1. Introduction

An increasing demand in the field of micro dispensing is the control of the delivered quantities of liquids. Especially non-contact dispensing applications in the pico- to the microliter range entail the need for novel measurement techniques to evaluate droplet volumes and the stability of the process in time. E.g., pharmaceutical research, based on quantification of several thousands of drug like substances or the imprint of quantitative readable lateral flow tests require precise information about the applied amount of liquids to improve the accuracy of the analysis [1–3]. Commonly, dispensing systems are calibrated by the manufacturer with test liquids e.g., water. This, however, does not guarantee the accuracy of each dispensed sample and implies trust in the ‘producers’ specifications and reported data. State of the art measurement techniques to determine droplet volumes in the nanoliter range are often not suitable for system integration due to their big mounting size or low accuracy, e.g., stroboscopic cameras [4]. Other approaches like gravimetric determination of the mass of a droplet require contact to the dispensed liquid which entails the loss of the measured liquid quantity. To address common shortcomings, non-contact measurement techniques have been reported. Based on the interaction of the dispensed liquid with an emitted light beam the droplet's presence as well as the dispensing process stability can be evaluated. However, these techniques do not allow for the measurement of a droplet's volume [5,6]. Alternative non-contact measurement methods are needed which enable the online determination of droplet properties like volume or velocity. Further requirements, like small size, easy system integration and a robust performance are also arising demands. A promising technique to address these challenges is to expose dispensed droplets to an electric field on their way from the orifice to the target. The change in the characteristics of the electric field measured by the change of the charge on a measurement electrode enables to evaluate properties like the droplet's volume and velocity [7,8]. This paper reports on a comprehensive simulation study of a free flying nanoliter droplet passing the electric field in between two charged electrodes with focus on the charge alternation induced by the droplet.

## 2. System Description

**Figure 1.**
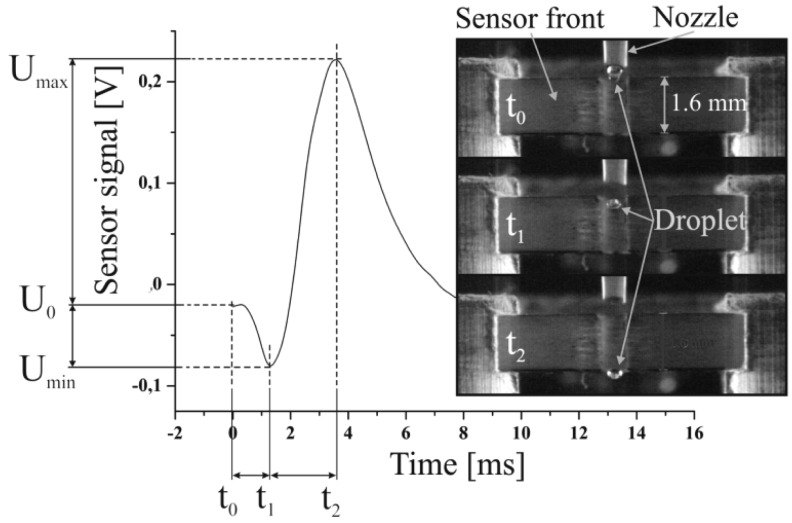
Previously achieved experimental signal characteristics correlated to droplet positions while a droplet is passing the capacitor.

The simulation study presented in this paper is based on measurement results achieved with a capacitive sensor prototype which was applied to detect single droplets in flight [8]. The used sensor provides an open plate capacitor forming the measurement electrodes, which are fabricated in PCB technology. A droplet which passes through the capacitor generates a specific time dependent capacity change which is amplified to a voltage signal like shown in Figure 1. The negative dip at the beginning of the signal describes unexpected signal characteristics which could not be explained in first instance. Even stroboscopic image analyses did not clarify the origin of this specific characteristic (see provided image sequence in Figure 1). The presented simulation study was initiated to investigate the characteristics of the electrical field causing the said specific signal. Furthermore, information about the effect of certain parameters influencing the electric field is aimed by this study.

**Figure 2.**
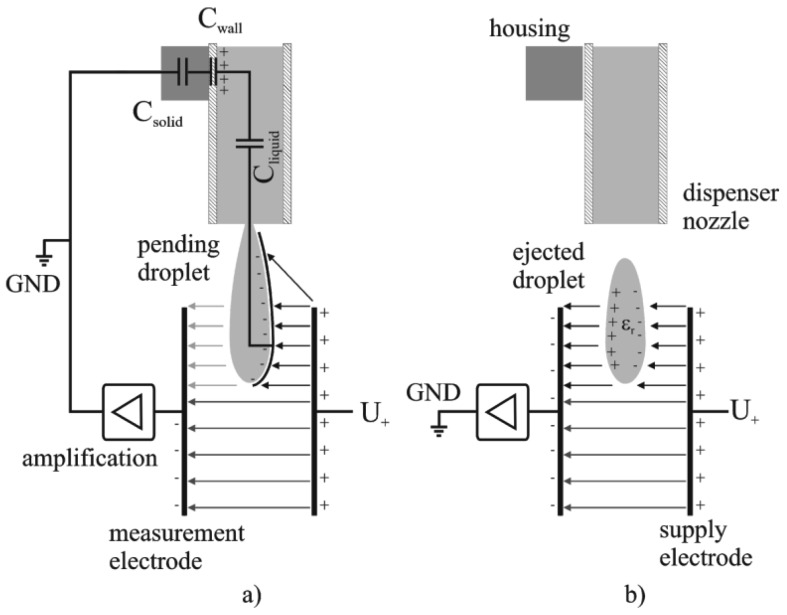
Two electrical equivalent circuits occurring during droplet ejection (**a**) a pending droplet is connected to the electronic system by capacitive coupling; (**b**) a droplet after tear-off acts as dielectric body.

A dispensing process comprises two distinct phases which are the droplet's growth until its tear-off from the dispenser nozzle and the free flight of the droplet after it has detached from the nozzle. It turns out that these two situations result in different boundary conditions for the electrical problem of the charged capacitor. Both situations can be described by two different electrical equivalent circuits like follows: in first consideration, a growing droplet is connected to the liquid inside the nozzle, which stays in contact to the aluminum housing of the used dispenser unit via the dispensing piston, like described in detail in [9]. The housing is electrically connected to the ground potential (GND = 0 V) of the electric read out circuit due to guarding reasons, to avoid the induction of external stray fields, caused by the dispenser actuation [8]. Though, the liquid is guided through a non-conductive polyimide tube, it still has to be supposed that the electrical potential of the liquid inside the nozzle is coupled to the housing potential via a capacitive network. This network can be considered to consist of a capacitor series connection given by C_liquid_, C_Wall_ and C_Solid_ as depicted in Figure 2. This effect is referred to in the following as “capacitive coupling” and it is essential for the specific signal characteristics. The signal recording starts when the dispenser is triggered and subsequently a growing droplet, which is still connected to the nozzle, is established. The charge on the positive electrode (U_+_ = 10 V) is compensated by negative free charge carriers, distributed on the measurement capacitor's negative electrode as well as on the increasing surface of the growing droplet. The increasing surface of the growing droplet leads to a shift from charge carriers from the negative electrode to the droplet surface and thus to an overall decrease of the charge on the negative electrode. Finally, this leads to a decrease of the capacity and explains the negative peak at the beginning of the signal characteristics like mentioned above. The appearing charge separation on the droplet's surface can be explained by the existence of free charge carriers, which are found even in de-ionized water based on the auto ionization of liquid water [10]. In principle this means, that beside the orientation polarization of the liquid molecule dipoles (considering water) initiated by the electric field, also a charge separation occurs, which compensates the applied electric field partly with regard to the GND potential. After the droplet's tear off from the nozzle the situation changes. The detached droplet is disconnected from the GND potential and acts as dielectric body only. A dielectric material changes the capacitance of a capacitor, according to its size and relative permittivity, and leads to an increase of the charge on the measurement electrode entailing a positive signal peak. As a conclusion the point of inflexion of the negative signal drop, see Figure 1, represents the point of droplet tear off from the dispenser nozzle, which could be used as a measure to evaluate e.g., the reproducibility of the droplet ejection process.

## 3. Numerical Model

### 3.1. Geometry

**Figure 3.**
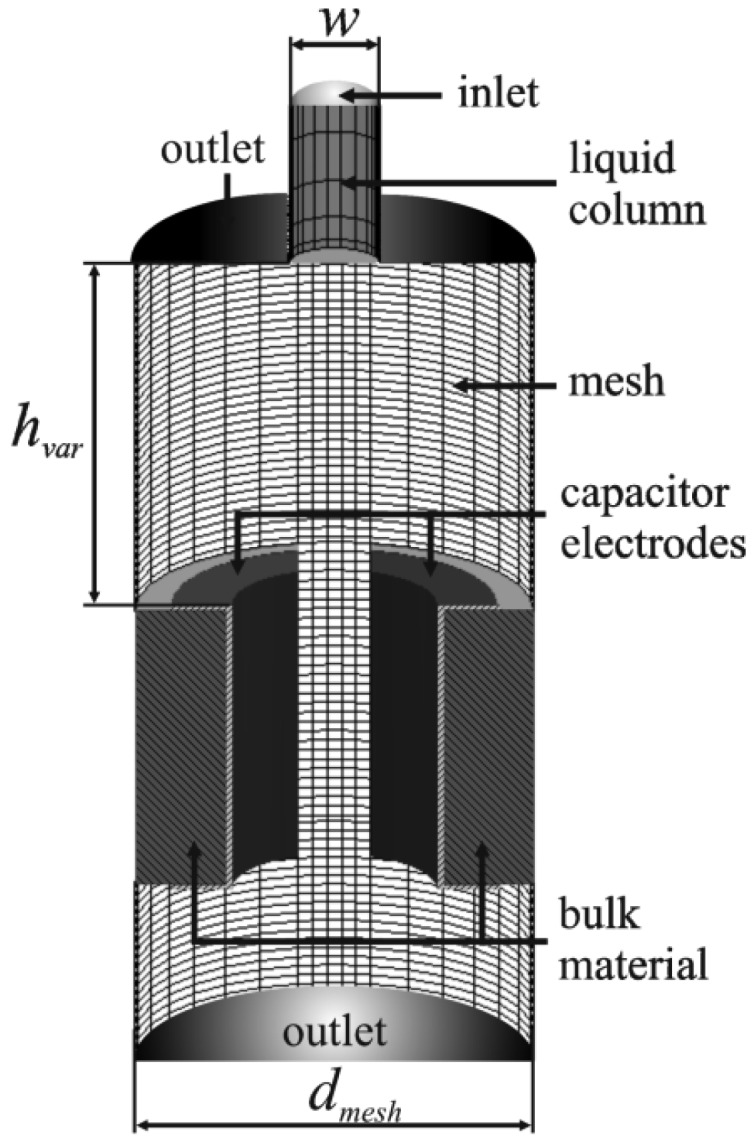
Computational domain as used for the CFD simulations.

The numerical multi-physics model to study the electrical field of the droplet sensor is based on a structured 3D grid consisting of the droplet generator, implemented by a liquid column, and the capacitor electrodes embedded in the sensor support material (bulk material). The computational domain, shown in Figure 3, consists mainly of half shell shaped electrodes, like used for the experimental realisation of the sensor prototype [9] and some air space above and below the capacitor. The liquid column at the top side of the domain represents the liquid phase inside the nozzle of a droplet generator and enables to simulate a droplet dispensing process. The nozzle diameter (w) is 500 μm corresponding to the experimentally used PipeJet™ dispensing system [9]. The variable nozzle length (l) enables to study the capacitive coupling effect in detail and was initially set to 500 μm. The capacitor electrodes are centred underneath the nozzle at a variable distance h_var_ with an initial height of 2 mm. The geometry of the capacitor featured an inner diameter (d) of 1.2 mm and a trench width (s) of 400 μm, separating the electrodes. The electrodes' height (z) set to 1.6 mm reflecting the prototype's geometry. The electrodes are embedded in bulk material made of FR4 (ε_r_ = 4.8). In order to save computation time the mirror symmetry of the problem has been exploited and only half of the spatial geometry was modelled as shown in Figure 3.

### 3.2. Governing Equations

The simulation of hydrodynamic fluid flows using CFD is based on the iterative solution of the discretized set of equations consisting of the basic physical conservation principles of mass, momentum and energy, given by the Navier Stokes Equations (NSE). The following sections introduce the basic equations required for the numerical calculation of the coupled simulation, considering the fluid dynamics of droplet ejection as well as the electrical interaction of a droplet and a capacitor.

#### 3.2.1. Finite Volume Method—FVM

The numerical simulation is based on the application of the finite volume method (FVM). The FVM is a numerical method for the solution of partial differential equations that calculates the values of the conserved variables across a considered volume. This, so called control volume, is defined by the discretization using a computational mesh. In case of incompressible Newtonian fluids considering the properties of an external electric field, which is the most appropriate for solving the described problem, the Navier Stokes momentum equation can be written as follows:

(1) $$\frac{\partial (\rho \vec{v})}{\partial t}+\nabla \cdot (\rho \vec{v}⊗\vec{v})=-\nabla P+\nabla \cdot (\mu \bar{\bar{\left(\nabla (\vec{v}\right)}}+\bar{\bar{\nabla {\left(\vec{v}\right)}^{T}}}))+{\vec{f}}^{el}+{\vec{f}}^{s}+\rho \vec{g}$$

The electrostatic force *f⃑^el^* is only implemented through a virtual force at the interface of the droplet due to the variation of the electric energy in this region. The electrostatic pressure force is not taken into account and not calculated in the simulations. Therefore, small discrepancies in the results might be repatriated to this fact. More detailed information about the FVM and the discretization of the NSE for numerical solution is provided in [11]. *f⃑^s^* is another source term in this equation and is notably used for the computation of the surface tension effect of the interface between air and water and to take into account the contact angle between water and the nozzle.

#### 3.2.2. Volume of Fluid—VOF

A further method that is required for simulation of the droplet generation is the volume of fluid method (VOF). The VOF method enables the simulation of two phase flows of immiscible fluids, which are in the considered case water and ambient air. The approach is based on the introduction of an additional field variable, the volume fraction *f*. The volume fraction *f* determines the phase distribution of the two phase flow by a value between 0 and 1 for each individual control volume. A completely liquid filled control volume is indicated by *f* = 1 whereas the volume fraction for the gas phase is *f* = 0. Any other state is continuously denoted by 0 < *f* < 1. The time propagation of the volume fraction *f* is calculated by the additionally solved passive transport Equation (2):

(2) $$\frac{\partial f}{\partial t}+\nabla \cdot (\vec{v}f)=0$$

Since this method determines the phase distribution in terms of the *f* value only, no discrete interfaces between the phases are described. Furthermore, the flow is calculated only for one “mixed” phase which is described by *f* values between 0 and 1, giving the ratio of the two phases in every control volume. Thus, also the fluid properties are considered as mean values like mean density, viscosity, *etc.* Any scalar property *x*_mean_ is averaged according the corresponding *f* value as follows:

(3) $$x_{\text{mean}}=(1-f)\cdot x_{\text{gas}}+f\cdot x_{\text{liquid}}$$

#### 3.2.3. Surface Reconstruction and Surface Tension

In order to model the surface tension of the liquid the pure VOF method like briefly described above is not sufficient. Furthermore, the surface tension has to be considered when dealing with capillary liquid flows like droplet ejection processes. The implementation of the surface tension to the numerical calculation is based on a surface reconstruction method to determine the curvature of the interface between the two phases. Here, the Piecewise Linear Interface Construction (PLIC) of 2nd order [12,13] is one of the most accurate method which was applied for the simulations, enabling the implementation of surface tension forces *γ* based on the Young-Laplace equation, given as:

(4) $$\gamma =\sigma k\hat{n}$$

where σ is the surface tension, k the curvature of the surface and *n̂* the surface unit normal vector [14]. The surface tension is implemented as source term *f⃑^s^* in the momentum equation to the FVM method, regarding the continuum surface force (CSF) model [15].

#### 3.2.4. Modelling of the Electrical Field

In addition to the fluid dynamics the electrical field of the capacitive transducer is modeled by the application of the electric module, provided by the CFD ACE+ tool [16]. This module enables the calculation of several different electrical effects based on the Maxwell equations. For the investigation of the considered problem, it is sufficient to consider the electrodynamic effects in the electrostatic limit only. The reason for this is that the occurring currents are very small and they occur on a large time scale only compared to the inductivities involved. Thus, electromagnetic effects can be neglected for the description of the charge redistribution on the capacitor electrodes. The electrostatic modeling is based on Gauss' Law which governs the fundamental basics of the topic. It states that the electric flux passing through a closed surface is equal to the charge enclosed by that surface, which can be written in differential form as follows:

(5) ∇⋅D=q

where *D* is the electric displacement flux density, given in (C/m^2^) and *q* is the volume charge density given in (C/m^3^). The relation of the electric flux density *D* and the electric field *E* is given by:

(6) D=ε⋅E

where *ε* = *ε_r_ε*_0_. The electric field vector *E* is non-rotational in the electrostatic limit (*i.e.*, ∇×*E*=0), thus a scalar electric potential can be defined by:

(7) E=−∇ψ

the insertion of Equations (5) and (7) in Equation (6) gives the Poisson's equation which is sufficient for the description of the electrostatic potential *ψ*, since the measured droplets are not in contact with any of the electrodes:

(8) $$\nabla \cdot (\varepsilon_{r}\varepsilon_{0}\cdot \nabla \psi )=-q$$

The electrostatic potential is then used as a source for coupling force at the interface of the droplet through the virtual force calculated by the CFD Ace+ software:

(9) $$F_{\text{virt}}=\nabla \left[\frac{1}{2}\varepsilon_{r}\varepsilon_{0}{\left|\nabla \psi \right|}^{2}\right]$$

The calculation of the total charge on an electrode by the electric module in a discrete way is implemented by:

(10) $$Q_{\psi_{0}}=\sum_{i=0}^{\text{face}\;\psi_{0}}\varepsilon_{i}E_{ni}A_{i}$$

which yields the sum of charge at each face with a fixed potential (*Ψ*_0_) of the incident displacement flux normal to the fixed electric potential boundary (*ε_i_E_ni_*) times the area of the face (A_i_). In the considered simulation the model Equation (10) is solved additionally to the fluid dynamic variables for each individual control volume by the finite volume method to calculate the charge on the capacitor electrodes.

### 3.3. Initial Conditions and Boundary Conditions

#### 3.3.1. Droplet Generation

**Figure 4.**
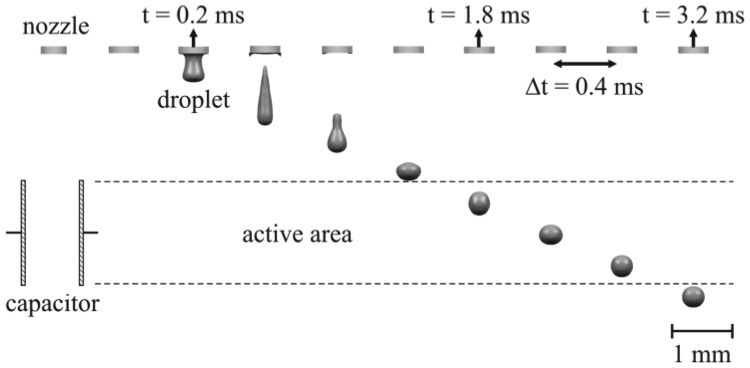
Simulated droplet ejection process applying the grid as described in Section 3.1. A flow (2.5 m/s) towards the nozzle is instantly stopped after 70 μs which initializes the droplet ejection.

The numerical study of the presented capacitive measurement method requires the implementation of a droplet ejection process which generates droplets with realistic properties in terms of shape, volume and velocity. To keep the focus on the electrostatic interaction, a simple model of a droplet ejection process was realised, based on a liquid flow boundary condition driving the droplet ejection. Therefore, a liquid flow of a constant velocity (v_flow_) of 2.5 m/s was set as boundary condition at the top inlet of the cylindrical liquid column (cf. Section 3.1). The flow was active for 70 μs and then stopped instantly to initiate the droplet tear off. The applied parameters led to a droplet ejection process like shown in Figure 4, which reflects realistic and representative droplet properties in volume (V = 33 nL), shape and velocity (v = 1.4 m/s). The wetting conditions for the walls, surrounding the nozzle were set to a wetting angle (α_water_) of 68° for pure water on the nozzle material. Furthermore, an outlet is defined at the lower end of the model to enable a balance of occurring pressure in the domain, caused by the droplet ejection. The wetting conditions at the electrodes are set to a ‘no wetting’ boundary condition (α_water_ = 180°), to avoid falsification of the results due to wet contamination of the electrodes.

#### 3.3.2. Electrical Model

The boundary condition for the electric field in between the capacitor electrodes is given by constant electrical potentials on the two opposite electrodes. The electrode on the left side (see Figure 3), is defined to be the measurement electrode and features an initial potential of 0 V (GND), whereas the electrode on the right side is set to a constant potential of 10 V. The sensor prototype described in [8] is supplied by an AC voltage of U_PP_ = 20 V at a frequency of 160 kHz. However, the simulation of one droplet measurement, considering the experimental conditions took more than one month. It was found that the DC boundary condition does not affect the results, but shortens the simulation time down to two days. Therefore, a DC supply was preferred for all simulations throughout this paper. The implementation of the capacitive coupling effect, like described in Section 2, is realised by an electrical boundary condition at the inlet of the liquid column (see Figure 3) which is set to GND potential. Therefore, the liquid column's height represents the overall capacitance described for the real device in Section 3.1 by the capacitive network consisting of C_liquid_, C_Wall_ and C_Solid_. To obtain quantitative results, the charge alternation on the measurement electrode is monitored during the simulations and extracted for further evaluation. The properties of the used fluids are taken from the material database, provided by CFD ACE+. The applied properties are listed in Table 1.

### 3.4. Solution Technique and Grid Refinement Study

**Figure 5.**
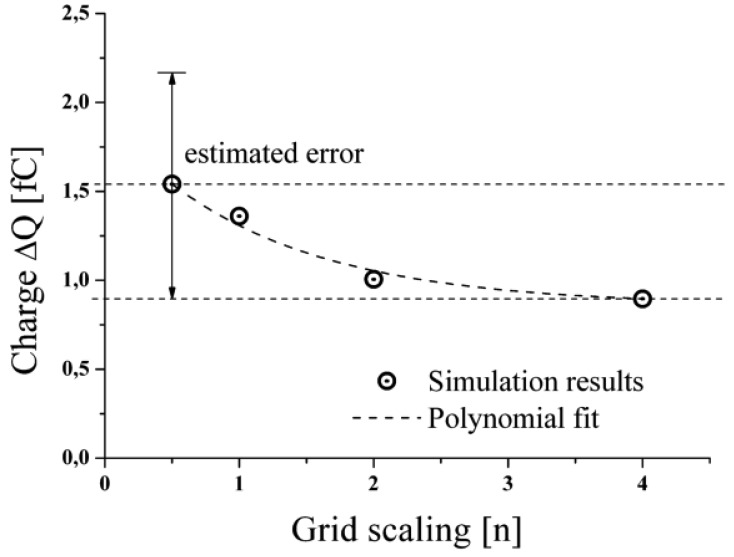
Results of the accomplished grid study to evaluate the accuracy of the used computational grid.

The applied solution technique follows the finite volume method, like described in Section 3.2, to solve the partial differential equations in the computational domain. The whole domain consists of 540,000 cells, whereas the area along the flight path of the droplet consists of a grid of smaller cells in comparison to the surrounding cells (cell size∼3:1). The planar geometry of the considered setup enabled to set a symmetry condition, which allowed for the calculation of only half of the real geometry, to save computation time. The simulation required transient conditions at a defined time step of Δt = 1 μs and a convergence criteria of 0.0001. To evaluate the accuracy of the numerical calculations, a brief grid refinement study was accomplished to investigate the influence of the cell size. To estimate the discretisation error the side length of all cells was decreased in all dimensions by a factor 2 and 4, as well as increased by a factor 2 respectively. Simulations were performed with the different grids by stationary simulation of an empty capacitor as well as for a capacitor with a droplet introduced in the middle of the electrodes at identical conditions like described above for the dynamic simulation model. The value of interest here was the change of the charge on the measurement electrode for the empty capacitor compared to the droplet filled one. The results are given in Figure 5 for the various grids.

It can be seen that the charge, depicted on the y-axis, declines for higher grid definition (x-axis). The polynomial fit converges to a value of about 1 fC representing the “real” physical condition. Based on this grid study the error of the transient simulations to be presented below was estimated to be about +29% if a grid of scaling 1 is applied. Though, this error is quite considerable, a grid or scaling 1 was applied for all further studies to hold the computational time within a reasonable time frame. Obviously, the presented grid study exhibits a convergence from larger to smaller values. However, in the general case it cannot be assumed that this is the case for any initial or boundary condition. Therefore, the estimated error should be assumed to be symmetric about the simulated values. Nevertheless, it turns out in all considered cases, like presented below, that the simulated results tend to overestimate the experimental or analytical findings by about approximately this error estimate of 29%.

## 4. Results and Discussion

### 4.1. Influence of Droplet Presence to the Electric Field

**Figure 6.**
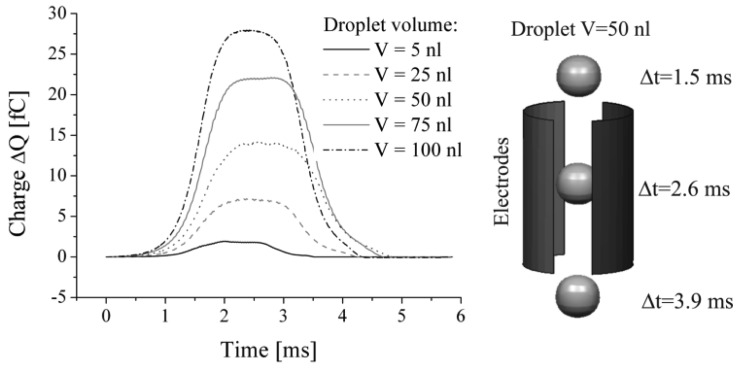
Change in charge as a function of time caused by spherically shaped droplets of different volumes passing the capacitor as shown in the illustration for a spherical droplet of V = 50 nL at three specific points in time.

To investigate the feasibility to solve the described multi-disciplinary problem the presented computational model was used in a simplified setup. Simulations were performed with defined spherical droplets of various volumes in the range from 5 to 100 nL neglecting the described droplet generation model. The droplets were defined as initial conditions in the model passing the capacitor with a constant shape at a defined velocity of v = 1 m/s. The charge characteristic on the measurement electrode was extracted as a function of time and is given in Figure 6 for five individual droplet sizes. The almost symmetric signals reflect the flight of the spherical droplets through the capacitor. The maximum change in charge is reached when the droplet approaches the horizontal centreline of the capacitor, like shown in Figure 6 (Δt = 2.6 ms at V = 50 nL). Like expected, the maximum charge alternation increases with higher droplet volumes, thus the feasibility to apply the sensor for volume measurements is confirmed. However, these simulations do not reflect the known signal characteristics, in particular the non-symmetric signal shape including the negative dip gained from experiments as displayed in Figure 1. Therefore, the full model of the dispensing process has to be considered to include the droplet tear off process as well as the effect of capacitive coupling.

### 4.2. Electric Field Characteristics Considering the Full Model

**Figure 7.**
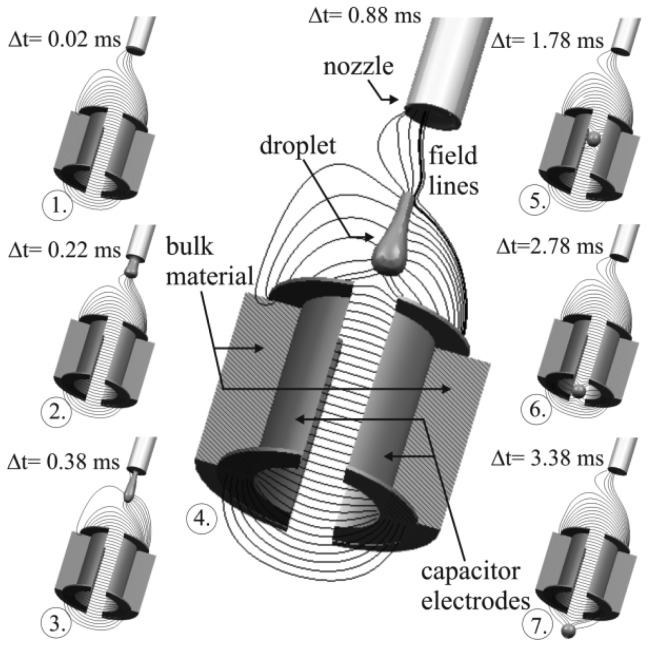
Simulated field distribution and droplet flight through the capacitor, including the droplet ejection process.

**Figure 8.**
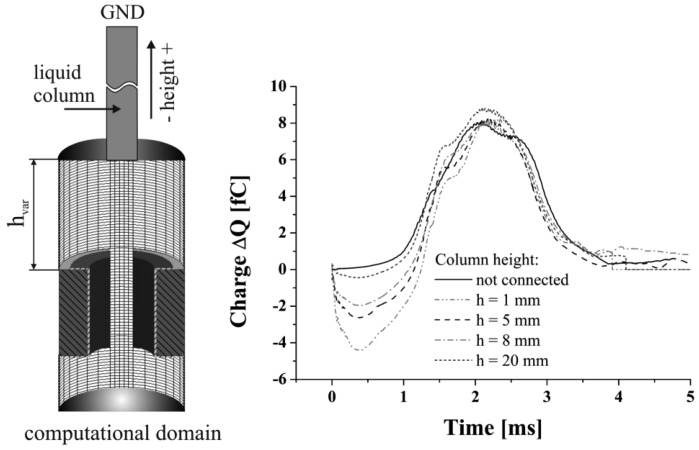
Investigation of the influence of the capacitive coupling effect by variations of the liquid column height.

The simulation of the full model as described in Section 3 results in different signal characteristics. Following the explanation given in Section 2, two separate situations can be distinguished, like depicted in Figure 7. The image shows a complete sequence of the simulated measurement process, given by 7 time discrete samples. It can be seen that the electric field gets increasingly attracted by the growing droplet from image 1 to 3 in Figure 7, leading to a declining charge on the measurement electrode (*cf.* Figure 8, line h = 5 mm which is the corresponding signal characteristics to this simulation). After the droplet's tear off (image 4 and following images.), the droplet acts as dielectric body, increasing the charge on the electrode due to its specific permittivity and volume. Some of the field lines still keep attracted by the liquid inside the nozzle, which is still connected to GND. This field however is constant over time, hence it does not influence the absolute value of the measurement. Movement of the liquid meniscus at the nozzle might entail additional fluctuations to the signal which could not be observed for the settings studied so far.

### 4.3. Capacitive Coupling

To confirm the influence of the capacitive coupling effect in a quantitative manner the height of the liquid column was varied, to increase the displacement of the ground potential at the end of the column, see Figure 8. This leads to a decrease of the liquid columns capacitance, which can be expected to increase the negative signal dip's amplitude. From the results given in Figure 8 can be taken that an increased height entails a decrease of the negative signal dip at a mostly constant maximum signal peak value. Considering an electrically disconnected boundary, omitting the ground potential at the end of the column, results in the elimination of the negative dip. A further effect caused by the changed columns height is the increase of the fluidic impedance leading to the generation of slightly different droplets. This might be the reason for the deviation in the maximum signal peaks. The minimum value of the negative signal dip is attained after 0.4 ms for all simulations, which reflects the tear-off of the droplet from the liquid column. These results lead to the conclusion that the described capacitive network presented in Section 2 reflects the electrical conditions of the presented system. A qualitative comparison to the experimentally gained signals, considering the ratio of negative peak value to positive peak value, led to the conclusion that a liquid column of h = 20 mm represents the experimental conditions best, which was applied for all further simulations.

### 4.4. Volume Effect

**Figure 9.**
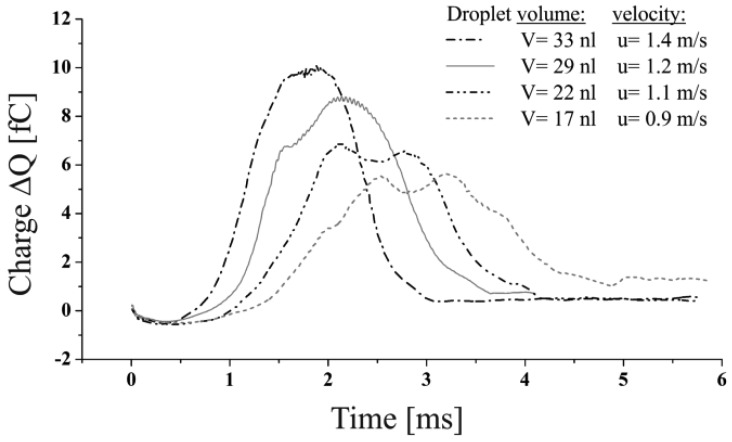
Charge alternation caused by dispensed droplets of different volumes and velocities.

The parameter of major interest is the effect of variable droplet volumes to the generated signals. A first proof of this influence was given in Section 4.1, Figure 6. However, these results were gained neglecting the influence of the dispensing process on the droplets shape as well as the effect of capacitive coupling. The generation of droplets of different volumes applying the described droplet ejection process required the adaption of the actuation time and velocity of the flow boundary condition like given in Table 2. Therefore, four different droplets could be generated featuring realistic parameters. The signals resulting from the simulation of the described droplets are given in Figure 9.

**Figure 10.**
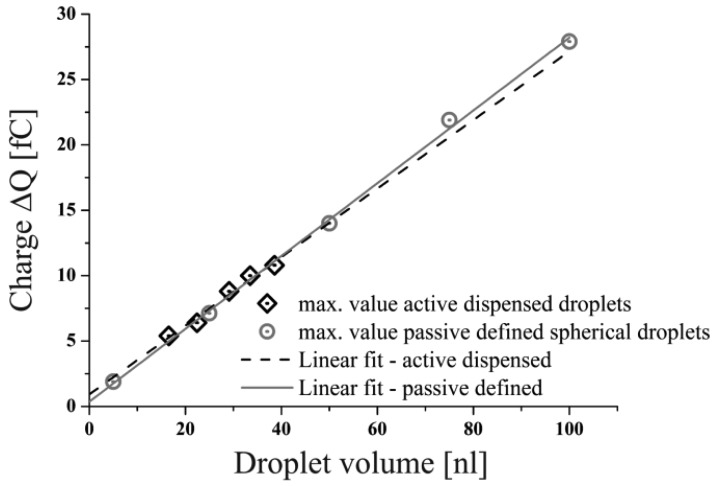
Correlation of droplet volume to the corresponding maximum change in charge for the spherical droplets as well as for the dispensed droplets.

It can be seen that the maximum change of the charge decreases with lower droplet volumes also in the full model simulation. A noticeable detail is the waved signal characteristics exhibited by the signals generated especially by smaller droplets (V = 22 nL and V = 17 nL, see Figure 9). Multiple signal maxima can be observed, which occur from fluctuations of the droplet shape while passing the capacitor. This effect implies the need of the identification of a value representing the realistic magnitude, equivalent to a spherical droplet of the causative volume. A good estimate for this is given by the mean value of the local minimum and its neighbouring maxima. In between the maximum lateral and longitudinal deformation of the droplet an almost spherical shape has to be attained which is represented by the said mean value. The applicability of this method was confirmed by the correlation of the calculated mean values to the results, gained from the simulation of the spherical droplets, shown in Section 4.1. The linear correlation of the droplet volumes to the signal peak values shown in Figure 10, enable to conclude that neither the influence of the capacitive coupling effect nor the deformation of the droplets caused by the ejection out of a nozzle does affect the measurement as far as the droplet volume—represented by the signal peak value—is concerned. Furthermore, the variable droplet velocity (see Table 2) seems not to affect the maximum change in charge for the considered velocity range (u_drop_ > 1.4 m/s).

### 4.5. Comparison with Experimental Results

To validate the established simulation model by experiment the maximum change in charge values taken from the simulation of spherical droplets were fed into an electrical network simulation representing the electrical amplification circuit of the capacitive sensor as described in [8]. The readout circuit is applied to transform the change in charge to a readable voltage level in the mV range. The electrical components, used for the simulation, featured the nominal values of the components used for the experiment. Thus, small tolerances which are inevitable for any electronic part are present in the experimental results but they are not reflected in the simulations.

**Figure 11.**
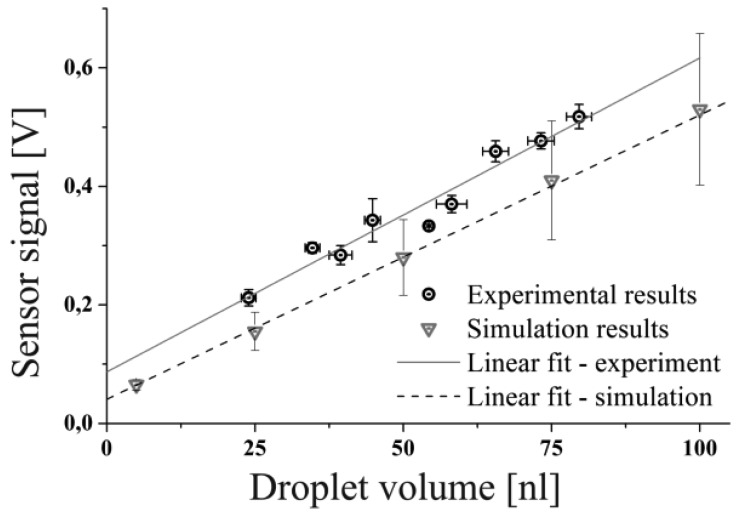
Comparison of the voltage signals obtained from the experiments and the results of the network simulation.

Figure 11 shows the comparison of the experimentally determined sensor peak signals for various droplet volumes in comparison to the peak voltages predicted by the network simulation. It can be seen that the sensitivity, given by the slope of the linear regressions are similar for both, the simulation and the experiment. Obviously, there is a slight offset between the measurement and the simulation which might be caused by deviations of the real values of the electronic components (e.g., resistance, capacity *etc.*) from the ideal values used for the simulation, influencing the total amplification of the electronic circuit. This offset could be probably explained or compensated by carefully checking each electronic component in the network model as well as the experimental setup for consistency, which is not the objective of this work. Another reason for the off-set might be the limited accuracy of the CFD simulations predicting the change of charge for a given droplet volume used as input to the network simulation as discussed before. Therefore, also the simulation results have to be furnished with error bars. The error bars given for the simulation results are gained from the presented grid refinement study, whereas the error bars for the experiment are deduced by the standard error of repeated measurements for each of the presented data points. Each data point represents approximately 60 single droplet measurements. In summary the results allow for the conclusion that the accomplished CFD simulation leads to a realistic linear scaling behavior and values for the change of the charge which are actually in the region of ΔQ < 30 fC for pure water droplets in the range of V < 100 nL. In combination with the Saber network model a complete numerical description of the experimental setup has been accomplished with reasonable accuracy and consistency with experimental results.

## 5. Conclusions

It has been shown that a multi-disciplinary simulation, comprising the simulation of a droplet ejection process and the interaction of the droplet with the electromagnetic field in the electrostatic limit can be successfully applied to quantitatively model the considered capacitive droplet sensor. The fundamental reason for the specific negative signal characteristics, known from previous experiments, could be explained by the effect of capacitive coupling. It was found that this effect can be used to identify the point of droplet tear-off from the nozzle, indicated by the negative signal peak. The study of variable droplet volumes has shown, that the change of the charge caused by the measured droplets follows a linear relation resulting in a sensitivity of S_i_ = 0.3 fC/nL. The verification of the CFD simulation results by comparison to the experimental results, considering the grid refinement study, confirmed that the presented models are correctly describing the considered problem. A final validation proved that the combination of the accomplished CFD simulations with an electrical network model enabled a complete numerical description of the experimental sensor prototype, including the electronic amplification circuit. In summary the established models and results can contribute significantly to the explanation and optimization of the capacitive measurement method. The presented capacitive droplet sensor can be considered as valuable contribution to the list of process control methods in the field of non-contact dispensing. In particular, the linear dependency of the change in charge on the volume of the causative droplet provides the basis for novel quantitative droplet sensors that are able to determine the volume of droplets with high accuracy in a non-contact manner. The application of such sensors can simplify the calibration and characterization of droplet generating devices as well as improve the quality of products, requiring small liquid quantities, by the application of online process control systems.

## Figures and Tables

**Table 1.** Material properties of the fluids used for the presented simulations.

**Physical property**	**DI water**	**air**
dynamic viscosity [mPas]	1.0	0.0185
permittivity	80.1 (f < GHz)	1
conductivity [1/Ωm]	5.5 × 10^−6^	1 × 10^−4^
Physical property	DI water	air
density [kg/m^3^]	1,000	1.161
Surface tension [N/m]	0.0725	–

**Table 2.** Droplet dispensing process actuation parameters and resulting droplet properties.

**Actuation Parameters**		**Droplet Properties**	
	
pulse duration [μs]	flow velocity [m/s]	volume [nL]	droplet velocity [m/s]
70	2.8	33	1.4
70	2.5	29	1.2
70	2.2	22	1.1
50	2.5	17	0.9
